# Syntheses and crystal structures of a new family of hybrid perovskites: C_5_H_14_N_2_·*A*Br_3_·0.5H_2_O (*A* = K, Rb, Cs)

**DOI:** 10.1107/S2056989019010338

**Published:** 2019-07-26

**Authors:** Sarah Ferrandin, Alexandra M. Z. Slawin, William T. A. Harrison

**Affiliations:** a Department of Chemistry, University of Aberdeen, Meston Walk, Aberdeen AB24 3UE, Scotland; b Department of Chemistry, University of St Andrews, KY16 9ST, Scotland

**Keywords:** crystal structure, hybrid perovskite, hydrogen bonding

## Abstract

The syntheses and crystal structures of three hybrid perovskites, *viz*. poly[1-methyl­piperizine-1,4-diium [tri-μ-bromido-potassium] hemihydrate], {(C_5_H_14_N_2_)[KBr_3_]·0.5H_2_O}_*n*_, (I), poly[1-methyl­piperizine-1,4-diium [tri-μ-bromido-rubidium] hemihydrate], {(C_5_H_14_N_2_)[RbBr_3_]·0.5H_2_O}_*n*_, (II), and poly[1-methyl­piperizine-1,4-diium [tri-μ-bromido-caesium] hemihydrate], {(C_5_H_14_N_2_)[CsBr_3_]·0.5H_2_O}_*n*_, (III), are described. These isostructural (space group *Amm*2) phases contain a three-dimensional, corner-sharing network of distorted *A*Br_6_ octa­hedra (*A* = K, Rb, Cs) with the same topology as the classical perovskite structure. The doubly protonated C_5_H_14_N_2_^2+^ cations occupy inter­stices bounded by eight octa­hedra and the water mol­ecules lie in square sites bounded by four octa­hedra. N—H⋯Br, N—H⋯(Br,Br), N—H⋯O and O—H⋯Br hydrogen bonds consolidate the structures.

## Abstract 2

The isostructural title compounds are a new family of hybrid perovskite hemihydrates.

## Chemical context   

Oxide perovskites of generic formula *AB*O_3_, where *A* and *B* are metal ions with a combined charge of +6, are probably the most-studied family of inorganic phases on account of their numerous physical properties and structural variety (Tilley, 2016 ▸). The aristotype (highest-possible symmetry) (Megaw, 1973 ▸) for this classic structure type is a three-dimensional network (space group *Pm* 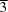 *m*) of undistorted, vertex-sharing, BO_6_ octa­hedra encapsulating the *A* cations in 12-coordinate dodeca­hedral cavities bounded by eight octa­hedra but lower symmetry (‘hettotype’) structures are very common, which can be systematically described in terms of tilting schemes of the octa­hedra (Woodward, 1997 ▸).

‘Hybrid’ *RMX*_3_ perovskites containing both inorganic and organic (mol­ecular) components have been studied intensively in the last few years due to their remarkable photo-voltaic and other optical properties (Xu *et al.*, 2019 ▸; Stylianakis *et al.*, 2019 ▸; Zuo *et al.*, 2019 ▸). The *R* ^+^ or *R* ^2+^ organic cation replaces the metallic *A* cation in an oxide perovskite and the *MX*_3_ (*X* = halide) octa­hedral network replaces the BO_3_ component of an oxide perovskite. Many of these studies have focused on lead halides [there are over 1400 papers on CH_3_NH_3_Pb*X*_3_ (*X* = Br, I) alone as of July 2019] and tin halides as the inorganic component of the structure (Stoumpos *et al.*, 2016 ▸) but other compositions are possible: several years ago, we described a family of alkali-metal–chloride perovskites templated by C_4_H_12_N_2_^2+^ piperizinium (or piperazin-1,4-diium) or C_6_H_14_N_2_^2+^ ‘dabconium’ (or 1,4-diazo­niabi­cyclo­[2.2.2]octa­ne) cations (Paton & Harrison, 2010 ▸). Notable features of these structures include the ‘inverse’ charges of the cations (*R* ^2+^ > *M* ^+^) compared to oxide perovskites, the inclusion of water mol­ecules of hydration in the C_4_H_12_N_2_·*A*Cl_3_·H_2_O (*A* = K, Rb, Cs) series and a novel chiral perovskite analogue (space group *P*3_2_21) for C_6_H_14_N_2_·RbCl_3_. This family has recently been extended by a number of phases (see *Database survey*) including C_6_H_14_N_2_·RbBr_3_ and C_7_H_16_N_2_·RbI_3_ (C_7_H_16_N_2_^2+^ = 1-methyl-1,4-di­aza­bicyclo­[2.2.2]octane-1,4-diium) (Zhang *et al.*, 2017 ▸), C_4_H_12_N_2_·RbBr_3_ (C_4_H_12_N_2_^2+^ = 3-ammonio­pyrrolidinium) (Pan *et al.*, 2017 ▸) and C_4_H_12_N_2_·NaI_3_ (Chen *et al.*, 2018 ▸), some of which have significant physical properties such as ferroelectricity.
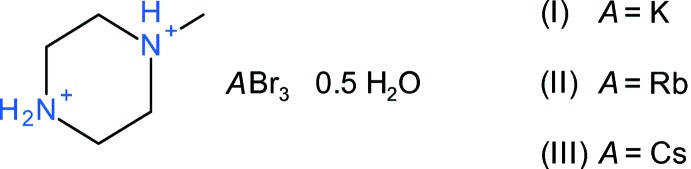


In this paper we describe the syntheses and structures of a new family of isostructural hybrid perovskite hemihydrates of formula C_5_H_14_N_2_·*A*Br_3_·0.5H_2_O (C_5_H_14_N_2_^2+^ = 1-methyl piperizinium cation) where *A* = K (I), Rb (II) and Cs (III).

## Structural commentary   

Compounds (I), (II) and (III) are isostructural as indicated by their ortho­rhom­bic unit cells, showing the expected trend of volume increase as a result of the increasing ionic radius (Shannon, 1976 ▸) of the alkali-metal cation on going from potassium (*r* = 1.52) to rubidium (*r* = 1.66) to caesium (*r* = 1.81 Å). This description will focus on the structure of (I) and note significant differences for (II) and (III) where applicable.

**Figure 1:**
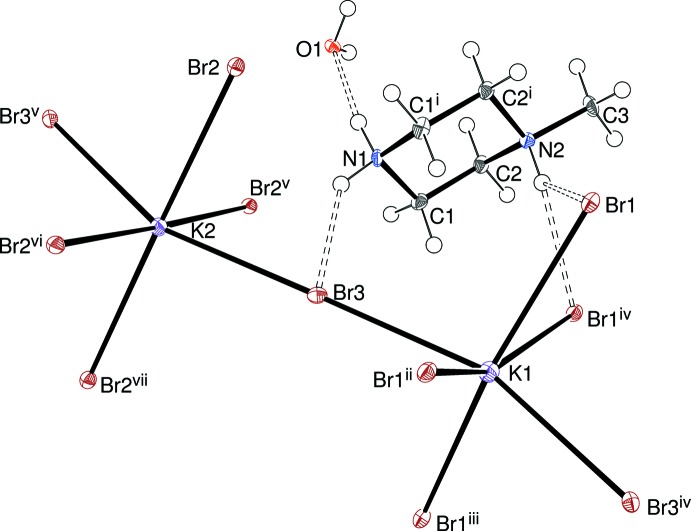
The asymmetric unit of (I) expanded to show the complete C_5_H_14_N_2_^2+^ cation, the complete potassium coordination polyhedra and the water mol­ecule (50% displacement ellipsoids). Symmetry codes: (i) *x*, 1 − *y*, *z*; (ii) 2 − *x*, 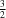 − *y*, 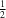 + *z*; (iii) *x*, *y* − 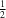, 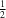 + *z*; (iv) 2 − *x*, 1 − *y*, *z*; (v) 1 − *x*, 1 − *y*, *z*; (vi) 1 − *x*, 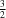 − *y*, 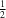 + *z*; (vii) *x*, *y* − 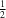, 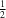 + *z*.

The asymmetric unit of (I) (Fig. 1 ▸) contains two methyl­ene groups (C1 and C2), an N1H_2_^+^ grouping, an N2H^+^ moiety and the carbon atom (C3) of a methyl group. N1, N2 and C3 lie on a (010) crystallographic mirror plane with *y* = ½ for the asymmetric atoms. The complete C_5_H_14_N_2_^2+^ cation is generated by the mirror to result in a typical (Dennington & Weller, 2018 ▸) chair conformation for this species with N1 and N2 deviating from the C1/C2/C1^i^/C2^i^ [symmetry code: (i) *x*, 1 – *y*, *z*] plane by −0.623 (7) and 0.708 (6) Å, respectively. The pendant C3 methyl group adopts an equatorial orientation with respect to the ring. A water mol­ecule with the O atom lying on the (½, ½, *z*) special position with *mm*2 symmetry (Wyckoff site 2*a*) is also present.

The inorganic component of the structure consists of two potassium ions, K1 (site symmetry *mm*2; Wyckoff site 2*b*) and K2 (*mm*2; 2*a*) and three bromide ions: Br1 [*m*(100); 4*e*], Br2 [*m*(100); 4*e*] and Br3 [*m*(010); 4*c*], which gives an overall inorganic stoichiometry of KBr_3_. Crystal symmetry constructs Br_6_ octa­hedra around each potassium ion and the mean K1—Br and K2—Br separations are 3.4770 and 3.3825 Å, respect­ively (Table 1 ▸); equivalent data for (II) (Table 2 ▸) are Rb1—Br = 3.4906 and Rb2—Br = 3.4194 Å; equivalent data for (III) (Table 3 ▸) are Cs1—Br = 3.5432 and Cs2—Br = 3.4780 Å. These data may be compared with the shortest K—Br and Rb—Br separations of 3.299 and 3.425 Å, respectively in the rocksalt-type KBr and RbBr structures and the Cs—Br separation of 3.716 Å in CsBr (eight-coordinate CsCl structure).

The K1 octa­hedron in (I) is considerably distorted with the smallest and largest *cis* Br—K—Br angles being 66.53 (4) and 110.57 (6)°, respectively and the *trans* angles spanning the range 157.98 (5)–160.13 (7)°. The K2 octa­hedron is more regular, with *cis* angles varying from 82.17 (3) to 97.55 (2)°. Two of the *trans* angles for K2 are close to 180° but the other is much smaller at 158.51 (7)°. The octa­hedral volume for the K1 octa­hedron is 53.2 Å^3^ and its angular variance (Robinson *et al.*, 1971 ▸) is 125.5°^2^. The equivalent data for the K2 octa­hedron are 50.7 Å^3^ and 45.0°^2^, respectively. The corresponding polyhedra in (II) and (III) are similarly distorted, with respective octa­hedral volumes and angular variances as follows: Rb1 53.7 Å^3^, 129.7°^2^; Rb2 52.1 Å^3^, 56.6°^2^; Cs1 55.8 Å^3^, 145.2°^2^; Cs2 54.2 Å^3^, 84.7°^2^.

Bond-valence-sum (BVS) calculations using the ‘extrapolated’ formalism of Brese & O’Keeffe (1991 ▸) give the following values in valence units: K1 0.66, K2 0.86, Rb1 0.88 Rb2 1.07, Cs1 1.21, Cs2 1.44 (expected value = 1.00 in all cases). These data suggest that K1 in (I) is considerably underbonded, which is consistent with the long mean K1—Br separation in (I) compared to the separation in KBr. Conversely, Cs2 in (III) is substanti­ally overbonded and must be a ‘tight fit’ in its octa­hedral site.

## Supra­molecular features   

**Figure 2:**
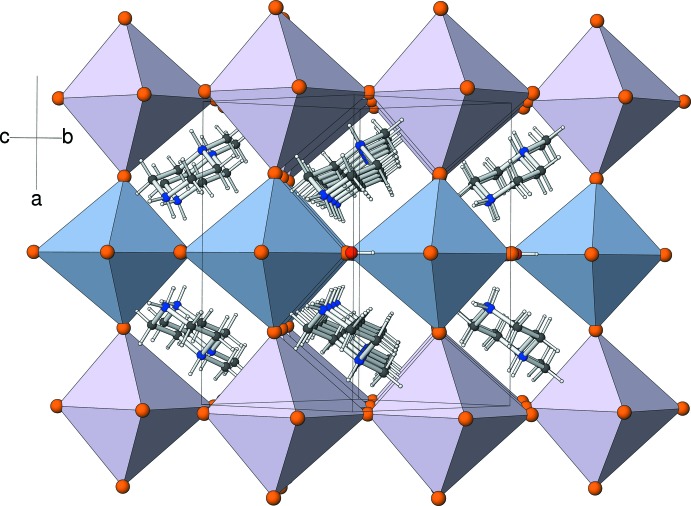
Polyhedral plot of the extended structure of (I) viewed down [011] with the K1Br_6_ octa­hedra coloured lilac and K2Br_6_ blue.

The linkage of the KBr_6_ octa­hedra in (I) in the *x*, *y* and *z* directions through their bromide-ion vertices leads to an infinite network of corner-sharing KBr_6_ octa­hedra akin to the network of BO_6_ octa­hedra in the classical *AB*O_3_ perovskite structure. Key features of the inorganic network are the K—Br—K bond angles (Table 1 ▸), with Br1 substanti­ally bent from the nominal linear bond [K1—Br1—K1^ii^ = 157.98 (5)°; symmetry code (ii) *x*, 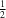 + *y*, *z* − 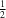], but Br2 and Br3 far less so. When the structure of (I) is viewed down [011], alternating (100) layers of K1- and K2-centred octa­hedra are apparent (Fig. 2 ▸). Within these (100) planes, the K1 atoms are linked by the Br1 ions and the K2 atoms are liked by the Br2 ions. Finally, Br3 provides the inter-layer linkages in the [100] direction.

**Figure 3:**
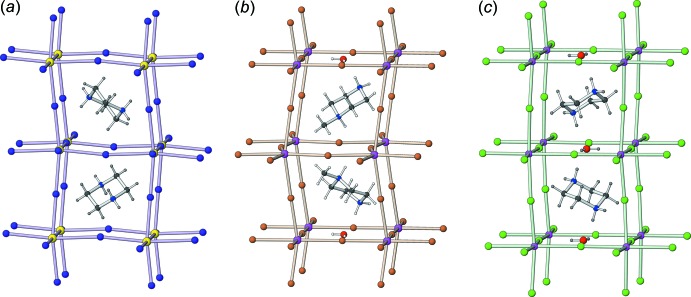
Comparison of the structures of (*a*) MEXMAG (redrawn from Chen *et al.*, 2018 ▸), (*b*) (I) and (*c*) GUYMIX (redrawn from Paton and Harrison, 2010 ▸). In MEXMAG, (I) and GUYMIX, the two octa­hedral cages shown are stacked in the [001], [100] and [001] directions, respectively. Note the alternation of water mol­ecules and empty sites in (I) with respect to the [100] direction whereas GUYMIX has a water mol­ecule in every square site in the [001] direction.

The 1-methyl­piperizinium cations occupy the central regions of the cages formed by eight KBr_6_ octa­hedra, obviously equivalent to the *A* cation site in a classical perovskite. The water mol­ecules lie at the centres of square sites bounded by four octa­hedra and stack in the [100] direction with alternating occupied and empty sites (see Fig. 3 ▸ and the *Database survey* section). Hydrogen bonding involving the encapsulated species is an important feature of the structure of (I): the N1H_2_^+^ group forms one N—H⋯Br3 link and one N—H⋯O link to the water mol­ecule (Table 4 ▸, Fig. 1 ▸) whereas the methyl­ated N2H^+^ group forms a rather long (and presumably weak) bifurcated N—H⋯(Br1,Br1) link. As just noted, the water mol­ecule accepts an N—H⋯O hydrogen bond from the organic cation and forms a pair of symmetry-equivalent O—H⋯Br2 hydrogen bonds. It is notable that *all* the C-bound H atoms in (I) are also potential hydrogen-bond donors to bromide ions based on the H⋯Br separations being significantly less than the van der Waals’ separation of 3.05 Å for these atoms. So far as the bromide ions are concerned, Br1 accepts one classical and three non-classical hydrogen bonds, Br2 accepts one classical and one non-classical and Br3 accepts one classical and two non-classical. The hydrogen-bonding schemes for (II) (Table 5 ▸) and (III) (Table 6 ▸) are essentially the same as that for (I).

## Database survey   

The title compounds and their significant analogue structures with their space groups and CCDC refcodes (Groom *et al.*, 2016 ▸) are listed in Table 7 ▸. These compounds now represent a significant family of hybrid perovskites featuring several different cations – the protonated forms of piperazine, dabco, 1-methyl­piperazine, 3-amino­pyrrolidine and ‘methyl dabco’ (1-methyl-1,4-di­aza­bicyclo­[2.2.2]octa­ne) – as well as different alkali metal cations and halide anions. The recently reported structure of MEXMAG (Chen *et al.*, 2108) has added sodium to the list of cations that can form these structures. Some structures such as HEJGOV (Zhang *et al.*, 2017 ▸) show notable physical properties such as ferroelectricity, which is of course a classic characteristic of oxide perovskites.

An inter­esting structural comparison may be made between MEXMAG (an ‘anhydrous’ *RAX*_3_ hybrid perovskite), (I) (an *RAX*_3_·0.5H_2_O hybrid perovskite hemihydrate) and GUYMIX (an *RAX*_3_·H_2_O hybrid perovskite hydrate) (Fig. 3 ▸). It may be seen that the pendant methyl groups of the C_5_H_14_N_2_^2+^ cations in (I) both point towards an empty square site and their steric bulk presumably prevents water mol­ecules from occupying that site. It is notable that the empty square site in (I) is associated with the reduced K1—Br1—K1 bond angles as noted above. Conversely, in MEXMAG, the iodide ions are perhaps too large to allow a water mol­ecule to fit between them and the piperazinium cation is forced to form long N—H⋯I hydrogen bonds (H⋯I = 3.14 Å) rather than N—H⋯O_w_ (w = water) links.

## Synthesis and crystallization   

To prepare (I), 0.3673 g (3.67 mmol) of 1-methyl piperazine and 0.4068 g (3.42 mmol) of KBr were added to 15.0 ml of 1.0 *M* aqueous HBr solution to result in a clear solution, which was left in a Petri dish to evaporate. After two or three days, colourless blocks of (I) were recovered, rinsed with acetone and dried in air. Compound (II) was prepared in the same way, with 0.4042 g (2.44 mmol) of RbBr replacing the KBr in (I) and (III) was prepared by using 0.4479 g (2.10 mmol) of CsBr in place of the KBr.

ATR–FTIR (cm^−1^) for (I) (selected): 3215*m* (NH_2_ asymmetric stretch), 2940*s* (NH_2_ symmetric stretch), 2692*s* (*sp* ^3^ C—H stretch), 1585*s* (NH_2_ bend) (assignments from Heacock & Marion, 1956 ▸); for (II) 3217*s*, 2998*s*, 2681*s*, 1548*s*; for (III) 3221*m*, 2995*s*, 2681*s*, 1548*s*. The IR spectra of (I), (II) and (III) are available in the supporting information.

## Refinement   

Crystal data, data collection and structure refinement details are summarized in Table 8 ▸. For each structure, the N- and C-bond hydrogen atoms were located geometrically (C—H = 0.98–0.99, N—H = 0.91–1.00Å) and refined as riding atoms. The water H atom was located in a difference map and refined as riding in its as-found relative position. The constraint *U*_iso_(H) = 1.2*U*_eq_(carrier) or 1.5*U*_eq_(methyl C) was applied in all cases.

## Supplementary Material

Crystal structure: contains datablock(s) I, II, III, global. DOI: 10.1107/S2056989019010338/vn2151sup1.cif

Structure factors: contains datablock(s) I. DOI: 10.1107/S2056989019010338/vn2151Isup2.hkl

Structure factors: contains datablock(s) II. DOI: 10.1107/S2056989019010338/vn2151IIsup3.hkl

Structure factors: contains datablock(s) III. DOI: 10.1107/S2056989019010338/vn2151IIIsup6.hkl

CCDC references: 1941726, 1941725, 1941724

Additional supporting information:  crystallographic information; 3D view; checkCIF report

## Figures and Tables

**Table 1:** Selected geometric parameters (Å, °) for (I)

K1—Br1^i^	3.4184 (13)	K2—Br2	3.4000 (16)
K1—Br1	3.5261 (18)	K2—Br2^ii^	3.4179 (15)
K1—Br3	3.4865 (10)	K2—Br3	3.3297 (10)
			
K1^iii^—Br1—K1	157.98 (5)	K2—Br3—K1	179.19 (5)
K2—Br2—K2^iii^	177.85 (5)		

Symmetry codes: (i) 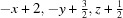; (ii) 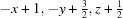; (iii) 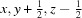.

**Table 2:** Selected geometric parameters (Å, °) for (II)

Rb1—Br1^i^	3.4639 (8)	Rb2—Br2	3.4326 (9)
Rb1—Br1	3.5323 (10)	Rb2—Br2^ii^	3.4336 (9)
Rb1—Br3	3.4756 (9)	Rb2—Br3	3.3919 (9)
			
Rb1^iii^—Br1—Rb1	157.67 (2)	Rb2—Br3—Rb1	178.12 (3)
Rb2—Br2—Rb2^iii^	176.93 (2)		

Symmetry codes: (i) 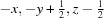; (ii) 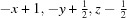; (iii) 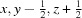.

**Table 3:** Selected geometric parameters (Å, °) for (III)

Cs1—Br1^i^	3.5319 (9)	Cs2—Br2	3.4923 (10)
Cs1—Br1	3.5873 (10)	Cs2—Br2^ii^	3.4790 (9)
Cs1—Br3	3.5105 (11)	Cs2—Br3	3.4627 (11)
			
Cs1^iii^—Br1—Cs1	156.07 (2)	Cs2—Br3—Cs1	175.99 (3)
Cs2^iii^—Br2—Cs2	174.97 (3)		

Symmetry codes: (i) 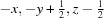; (ii) 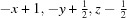; (iii) 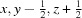.

**Table 4:** Hydrogen-bond geometry (Å, °) for (I)

*D*—H⋯*A*	*D*—H	H⋯*A*	*D*⋯*A*	*D*—H⋯*A*
N1—H2*N*⋯O1	0.91	1.89	2.799 (5)	172
N1—H1*N*⋯Br3	0.91	2.47	3.232 (5)	141
N2—H3*N*⋯Br1	1.00	2.67	3.438 (4)	133
N2—H3*N*⋯Br1^iv^	1.00	2.67	3.438 (4)	133
O1—H1*O*⋯Br2^v^	0.87	2.34	3.200 (3)	172
C1—H1*A*⋯Br2^vi^	0.99	2.88	3.614 (4)	131
C1—H1*B*⋯Br1^iv^	0.99	3.00	3.703 (4)	129
C2—H2*B*⋯Br1^iv^	0.99	3.05	3.517 (4)	111
C2—H2*B*⋯Br3^v^	0.99	2.82	3.525 (4)	129
C3—H3*A*⋯Br1^vii^	0.98	2.96	3.857 (4)	153
C3—H3*B*⋯Br3^viii^	0.98	2.75	3.615 (6)	148

Symmetry codes: (iv) 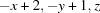; (v) 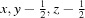; (vi) 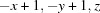; (vii) 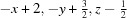; (viii) 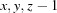.

**Table 5:** Hydrogen-bond geometry (Å, °) for (II)

*D*—H⋯*A*	*D*—H	H⋯*A*	*D*⋯*A*	*D*—H⋯*A*
N1—H1*N*⋯O1	0.91	1.91	2.816 (5)	173
N1—H2*N*⋯Br3	0.91	2.49	3.243 (4)	140
N2—H3*N*⋯Br1	1.00	2.68	3.448 (4)	134
N2—H3*N*⋯Br1^iv^	1.00	2.68	3.448 (4)	134
O1—H1*O*⋯Br2^v^	0.87	2.34	3.212 (3)	173
C1—H1*A*⋯Br1^iv^	0.99	3.01	3.717 (4)	129
C1—H1*B*⋯Br2^vi^	0.99	2.92	3.652 (4)	131
C2—H2*B*⋯Br1^iv^	0.99	3.06	3.531 (4)	111
C2—H2*B*⋯Br3^v^	0.99	2.85	3.560 (4)	130
C3—H3*A*⋯Br1^vii^	0.99	3.03	3.927 (4)	152
C3—H3*B*⋯Br3^viii^	0.99	2.79	3.653 (5)	146

Symmetry codes: (iv) 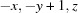; (v) 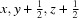; (vi) 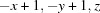; (vii) 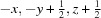; (viii) 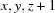.

**Table 6:** Hydrogen-bond geometry (Å, °) for (III)

*D*—H⋯*A*	*D*—H	H⋯*A*	*D*⋯*A*	*D*—H⋯*A*
N1—H2*N*⋯O1	0.91	1.94	2.845 (7)	173
N1—H1*N*⋯Br3	0.91	2.51	3.259 (6)	140
N2—H3*N*⋯Br1	1.00	2.68	3.446 (5)	134
N2—H3*N*⋯Br1^iv^	1.00	2.68	3.446 (5)	134
O1—H1*O*⋯Br2^v^	0.89	2.36	3.242 (4)	175
C1—H1*A*⋯Br1^iv^	0.99	3.07	3.762 (5)	128
C1—H1*B*⋯Br2^vi^	0.99	2.99	3.712 (5)	131
C2—H2*B*⋯Br1^iv^	0.99	3.08	3.550 (5)	111
C2—H2*B*⋯Br3^v^	0.99	2.92	3.643 (4)	131
C3—H3*B*⋯Br3^vii^	1.00	2.86	3.726 (7)	145

Symmetry codes: (iv) 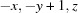; (v) 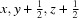; (vi) 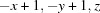; (vii) 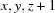.

**Table 7:** Summary of hybrid perovskite structures based on *AX*_3_ alkali-metal–halide octa­hedral networks

Code/refcode	Formula	Space group	Reference
(I)	C_5_H_14_N_2_·KBr_3_·0.5H_2_O	*Amm*2	This work
(II)	C_5_H_14_N_2_·RbBr_3_·0.5H_2_O	*Amm*2	This work
(III)	C_5_H_14_N_2_·CsBr_3_·0.5H_2_O	*Amm*2	This work
GUYMIX	C_4_H_12_N_2_·KCl_3_·H_2_O	*Pbcm*	Paton & Harrison (2010 ▸)
GUYMOD	C_4_H_12_N_2_·RbCl_3_·H_2_O	*Pbcm*	Paton & Harrison (2010 ▸)
GUYMUJ	C_4_H_12_N_2_·CsCl_3_·H_2_O	*Pbcm*	Paton & Harrison (2010 ▸)
MOMLEI	C_4_H_12_N_2_·KBr_3_·H_2_O	*Pbcm*	Harrison (2019*a* ▸)
MOMSEP	C_4_H_12_N_2_·RbBr_3_·H_2_O	*Pbcm*	Harrison (2019*b* ▸)
FIZYIZ	C_6_H_14_N_2_·KBr_3_	*P*3_1_21	Hongzhang (2019 ▸)
GUYNEU	C_6_H_14_N_2_·RbCl_3_	*P*3_2_21	Paton & Harrison (2010 ▸)
HEJGUB	C_6_H_14_N_2_·RbBr_3_	*P*3_2_21	Zhang *et al.* (2017 ▸)
GUYNEU02^*a*^	C_6_H_14_N_2_·RbCl_3_	*Pm* 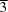 *m*	Zhang *et al.* (2017 ▸)
HEJGUB01^*a*^	C_6_H_14_N_2_·RbBr_3_	*Pm* 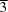 *m*	Zhang *et al.* (2017 ▸)
GUYNIY	C_6_H_14_N_2_·CsCl_3_	*C*2/*c*	Paton & Harrison (2010 ▸)
HEJGOV	C_7_H_16_N_2_·RbI_3_	*P*432	Zhang *et al.* (2017 ▸)
HEJGOV01	C_7_H_16_N_2_·RbI_3_	*R*3	Zhang *et al.* (2017 ▸)
GEFLOV	C_4_H_12_N_2_·RbBr_3_	*I*a	Pan *et al.* (2017 ▸)
GEFLOV01^*a*^	C_4_H_12_N_2_·RbBr_3_	*Pm* 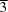 *m*	Pan *et al.* (2017 ▸)
MEXMAG	C_4_H_12_N_2_·NaI_3_	*C*2/*c*	Chen *et al.* (2018 ▸)

Redetermined structures not included. Note: (*a*) high-temperature polymorph.

**Table 8:** Experimental details

	(I)	(II)	(III)
Crystal data			
Chemical formula	(C_5_H_14_N_2_)[KBr_3_]·0.5H_2_O	(C_5_H_14_N_2_)[RbBr_3_]·0.5H_2_O	(C_5_H_14_N_2_)[CsBr_3_]·0.5H_2_O
*M*_r_	390.02	436.39	483.83
Crystal system, space group	Orthorhombic, *A* *m* *m*2	Orthorhombic, *A* *m* *m*2	Orthorhombic, *A* *m* *m*2
Temperature (K)	93	93	93
*a*, *b*, *c* (Å)	13.411 (3), 9.488 (2), 9.790 (2)	13.477 (3), 9.5617 (19), 9.850 (2)	13.610 (3), 9.7201 (19), 9.977 (2)
*V* (Å^3^)	1245.7 (5)	1269.3 (5)	1319.9 (5)
*Z*	4	4	4
Radiation type	Mo *K*α	Mo *K*α	Mo *K*α
μ (mm^−1^)	10.01	13.31	11.85
Crystal size (mm)	0.10 × 0.08 × 0.08	0.15 × 0.10 × 0.10	0.10 × 0.10 × 0.10

Data collection			
Diffractometer	Rigaku Pilatus 200K CCD	Rigaku Pilatus 200K CCD	Rigaku Pilatus 200K CCD
Absorption correction	Multi-scan (*CrysAlis PRO*; Rigaku, 2017 ▸)	Multi-scan (*CrysAlis PRO*; Rigaku, 2017 ▸)	Multi-scan (*CrysAlis PRO*; Rigaku, 2017 ▸)
*T*_min_, *T*_max_	0.406, 1.000	0.347, 1.000	0.463, 1.000
No. of measured, independent and observed [*I* > 2σ(*I*)] reflections	3890, 1235, 1208	6851, 1264, 1244	3718, 1312, 1293
*R*_int_	0.033	0.037	0.022
(sin θ/λ)_max_ (Å^−1^)	0.602	0.602	0.603

Refinement			
*R*[*F* ^2^ > 2σ(*F* ^2^)], *wR*(*F* ^2^), *S*	0.015, 0.031, 0.90	0.013, 0.028, 1.01	0.013, 0.029, 0.94
No. of reflections	1235	1264	1312
No. of parameters	68	69	68
No. of restraints	1	1	1
H-atom treatment	H-atom parameters constrained	H-atom parameters constrained	H-atom parameters constrained
Δρ_max_, Δρ_min_ (e Å^−3^)	0.41, −0.36	0.27, −0.35	0.58, −0.70
Absolute structure	Parsons *et al.* (2013 ▸)	Parsons *et al.* (2013 ▸)	Parsons *et al.* (2013 ▸)
Absolute structure parameter	−0.001 (11)	−0.001 (13)	0.016 (7)

Computer programs: *CrysAlis PRO* (Rigaku, 2017 ▸), *SHELXS7* (Sheldrick, 2008 ▸), *SHELXL2014/7* (Sheldrick, 2015 ▸), *ORTEP-3 for Windows* (Farrugia, 2012 ▸) and *publCIF* (Westrip, 2010 ▸).

## supplementary crystallographic information

### Poly[1-methylpiperizine-1,4-diium [tri-µ-bromido-potassium] hemihydrate] (I) Crystal data

**Table:**

(C_5_H_14_N_2_)[KBr_3_]·0.5H_2_O	*D*_x_ = 2.080 Mg m^−^^3^
*M**_r_* = 390.02	Mo *K*α radiation, λ = 0.71073 Å
Orthorhombic, *A**m**m*2	Cell parameters from 2257 reflections
*a* = 13.411 (3) Å	θ = 3.0–27.2°
*b* = 9.488 (2) Å	µ = 10.01 mm^−^^1^
*c* = 9.790 (2) Å	*T* = 93 K
*V* = 1245.7 (5) Å^3^	Prism, colourless
*Z* = 4	0.10 × 0.08 × 0.08 mm
*F*(000) = 748	

### Poly[1-methylpiperizine-1,4-diium [tri-µ-bromido-potassium] hemihydrate] (I) Data collection

**Table:**

Rigaku Pilatus 200K CCD diffractometer	1208 reflections with *I* > 2σ(*I*)
ω scans	*R*_int_ = 0.033
Absorption correction: multi-scan (CrysalisPro; Rigaku, 2017)	θ_max_ = 25.3°, θ_min_ = 3.0°
*T*_min_ = 0.406, *T*_max_ = 1.000	*h* = −16→16
3890 measured reflections	*k* = −11→11
1235 independent reflections	*l* = −11→11

### Poly[1-methylpiperizine-1,4-diium [tri-µ-bromido-potassium] hemihydrate] (I) Refinement

**Table:**

Refinement on *F*^2^	H-atom parameters constrained
Least-squares matrix: full	*w* = 1/[σ^2^(*F*_o_^2^)] where *P* = (*F*_o_^2^ + 2*F*_c_^2^)/3
*R*[*F*^2^ > 2σ(*F*^2^)] = 0.015	(Δ/σ)_max_ < 0.001
*wR*(*F*^2^) = 0.031	Δρ_max_ = 0.41 e Å^−^^3^
*S* = 0.90	Δρ_min_ = −0.36 e Å^−^^3^
1235 reflections	Extinction correction: SHELXL-2014/7 (Sheldrick 2015), Fc^*^=kFc[1+0.001xFc^2^λ^3^/sin(2θ)]^-1/4^
68 parameters	Extinction coefficient: 0.00195 (16)
1 restraint	Absolute structure: Parsons *et al.* (2013)
Primary atom site location: structure-invariant direct methods	Absolute structure parameter: −0.001 (11)
Hydrogen site location: mixed	

### Poly[1-methylpiperizine-1,4-diium [tri-µ-bromido-potassium] hemihydrate] (I) Special details

**Table:**

Geometry. All esds (except the esd in the dihedral angle between two l.s. planes) are estimated using the full covariance matrix. The cell esds are taken into account individually in the estimation of esds in distances, angles and torsion angles; correlations between esds in cell parameters are only used when they are defined by crystal symmetry. An approximate (isotropic) treatment of cell esds is used for estimating esds involving l.s. planes.

### Poly[1-methylpiperizine-1,4-diium [tri-µ-bromido-potassium] hemihydrate] (I) Fractional atomic coordinates and isotropic or equivalent isotropic displacement parameters (Å^2^)

**Table:**

	*x*	*y*	*z*	*U*_iso_*/*U*_eq_	
K1	1.0000	0.5000	0.9058 (2)	0.0145 (4)	
K2	0.5000	0.5000	1.0306 (2)	0.0120 (3)	
Br1	1.0000	0.70384 (5)	0.60463 (6)	0.01165 (14)	
Br2	0.5000	0.74449 (6)	0.77675 (6)	0.01087 (14)	
Br3	0.74393 (4)	0.5000	0.96724 (6)	0.01101 (14)	
C1	0.7289 (3)	0.3693 (4)	0.6163 (5)	0.0123 (9)	
H1A	0.6861	0.2859	0.6321	0.015*	
H1B	0.7878	0.3614	0.6767	0.015*	
C2	0.7626 (3)	0.3706 (4)	0.4700 (5)	0.0107 (8)	
H2A	0.7038	0.3696	0.4089	0.013*	
H2B	0.8027	0.2851	0.4510	0.013*	
C3	0.8629 (4)	0.5000	0.2985 (6)	0.0145 (12)	
H3A	0.9028	0.5851	0.2831	0.022*	
H3B	0.8067	0.5000	0.2346	0.022*	
N1	0.6720 (3)	0.5000	0.6522 (5)	0.0098 (10)	
H1N	0.6586	0.5000	0.7433	0.012*	
H2N	0.6128	0.5000	0.6067	0.012*	
N2	0.8244 (3)	0.5000	0.4421 (5)	0.0091 (10)	
H3N	0.8827	0.5000	0.5059	0.011*	
O1	0.5000	0.5000	0.4902 (5)	0.0088 (12)	
H1O	0.5000	0.4251	0.4392	0.011*	

### Poly[1-methylpiperizine-1,4-diium [tri-µ-bromido-potassium] hemihydrate] (I) Atomic displacement parameters (Å^2^)

**Table:**

	*U*^11^	*U*^22^	*U*^33^	*U*^12^	*U*^13^	*U*^23^
K1	0.0132 (9)	0.0147 (8)	0.0156 (10)	0.000	0.000	0.000
K2	0.0105 (8)	0.0138 (8)	0.0117 (9)	0.000	0.000	0.000
Br1	0.0086 (3)	0.0111 (3)	0.0152 (3)	0.000	0.000	−0.0018 (2)
Br2	0.0102 (3)	0.0114 (3)	0.0110 (3)	0.000	0.000	−0.0015 (2)
Br3	0.0119 (3)	0.0102 (3)	0.0110 (3)	0.000	−0.0026 (2)	0.000
C1	0.0105 (17)	0.0092 (18)	0.017 (2)	0.0022 (14)	0.0017 (18)	0.0032 (19)
C2	0.0098 (18)	0.0087 (17)	0.014 (2)	−0.0005 (14)	0.0021 (19)	0.000 (2)
C3	0.017 (3)	0.019 (3)	0.008 (3)	0.000	0.005 (3)	0.000
N1	0.007 (2)	0.014 (2)	0.008 (3)	0.000	0.0026 (19)	0.000
N2	0.009 (2)	0.009 (2)	0.010 (3)	0.000	0.0003 (19)	0.000
O1	0.012 (2)	0.008 (2)	0.007 (3)	0.000	0.000	0.000

### Poly[1-methylpiperizine-1,4-diium [tri-µ-bromido-potassium] hemihydrate] (I) Geometric parameters (Å, º)

**Table:**

K1—Br1^i^	3.4184 (13)	C1—C2	1.502 (6)
K1—Br1^ii^	3.4184 (13)	C1—H1A	0.9900
K1—Br1^iii^	3.5261 (18)	C1—H1B	0.9900
K1—Br1	3.5261 (18)	C2—N2	1.506 (4)
K1—Br3	3.4865 (10)	C2—H2A	0.9900
K1—Br3^iii^	3.4865 (10)	C2—H2B	0.9900
K2—Br2^iv^	3.4000 (16)	C3—N2	1.497 (7)
K2—Br2	3.4000 (16)	C3—H3A	0.9800
K2—Br2^v^	3.4179 (15)	C3—H3B	0.9799
K2—Br2^ii^	3.4179 (15)	N1—C1^vii^	1.498 (4)
K2—Br3^iv^	3.3297 (10)	N1—H1N	0.9100
K2—Br3	3.3297 (10)	N1—H2N	0.9100
Br1—K1^vi^	3.4183 (13)	N2—C2^vii^	1.506 (4)
Br2—K2^vi^	3.4179 (15)	N2—H3N	1.0000
C1—N1	1.498 (4)	O1—H1O	0.8686
			
Br1^i^—K1—Br1^ii^	110.57 (6)	K1^vi^—Br1—K1	157.98 (5)
Br1^i^—K1—Br3	84.36 (2)	K2—Br2—K2^vi^	177.85 (5)
Br1^ii^—K1—Br3	84.36 (2)	K2—Br3—K1	179.19 (5)
Br1^i^—K1—Br3^iii^	84.36 (2)	N1—C1—C2	111.8 (3)
Br1^ii^—K1—Br3^iii^	84.36 (2)	N1—C1—H1A	109.3
Br3—K1—Br3^iii^	160.13 (7)	C2—C1—H1A	109.3
Br1^i^—K1—Br1^iii^	157.98 (5)	N1—C1—H1B	109.3
Br1^ii^—K1—Br1^iii^	91.449 (19)	C2—C1—H1B	109.3
Br3—K1—Br1^iii^	98.30 (3)	H1A—C1—H1B	107.9
Br3^iii^—K1—Br1^iii^	98.30 (3)	C1—C2—N2	110.2 (3)
Br1^i^—K1—Br1	91.449 (18)	C1—C2—H2A	109.6
Br1^ii^—K1—Br1	157.98 (5)	N2—C2—H2A	109.6
Br3—K1—Br1	98.30 (3)	C1—C2—H2B	109.6
Br3^iii^—K1—Br1	98.30 (3)	N2—C2—H2B	109.6
Br1^iii^—K1—Br1	66.53 (4)	H2A—C2—H2B	108.1
Br3^iv^—K2—Br3	158.51 (7)	N2—C3—H3A	109.4
Br3^iv^—K2—Br2^iv^	82.17 (3)	N2—C3—H3B	109.5
Br3—K2—Br2^iv^	82.17 (3)	H3A—C3—H3B	108.7
Br3^iv^—K2—Br2	82.17 (3)	C1—N1—C1^vii^	111.7 (4)
Br3—K2—Br2	82.17 (3)	C1—N1—H1N	109.3
Br2^iv^—K2—Br2	86.05 (5)	C1^vii^—N1—H1N	109.3
Br3^iv^—K2—Br2^v^	97.55 (2)	C1—N1—H2N	109.3
Br3—K2—Br2^v^	97.55 (2)	C1^vii^—N1—H2N	109.3
Br2^iv^—K2—Br2^v^	177.85 (5)	H1N—N1—H2N	107.9
Br2—K2—Br2^v^	91.800 (17)	C3—N2—C2^vii^	111.1 (3)
Br3^iv^—K2—Br2^ii^	97.55 (2)	C3—N2—C2	111.1 (3)
Br3—K2—Br2^ii^	97.55 (2)	C2^vii^—N2—C2	109.2 (4)
Br2^iv^—K2—Br2^ii^	91.800 (17)	C3—N2—H3N	108.4
Br2—K2—Br2^ii^	177.85 (5)	C2^vii^—N2—H3N	108.4
Br2^v^—K2—Br2^ii^	90.35 (5)	C2—N2—H3N	108.4
			
N1—C1—C2—N2	56.6 (4)	C1—C2—N2—C3	177.3 (3)
C2—C1—N1—C1^vii^	−52.9 (5)	C1—C2—N2—C2^vii^	−59.8 (5)

Symmetry codes: (i) −*x*+2, −*y*+3/2, *z*+1/2; (ii) *x*, *y*−1/2, *z*+1/2; (iii) −*x*+2, −*y*+1, *z*; (iv) −*x*+1, −*y*+1, *z*; (v) −*x*+1, −*y*+3/2, *z*+1/2; (vi) *x*, *y*+1/2, *z*−1/2; (vii) *x*, −*y*+1, *z*.

### Poly[1-methylpiperizine-1,4-diium [tri-µ-bromido-potassium] hemihydrate] (I) Hydrogen-bond geometry (Å, º)

**Table:**

*D*—H···*A*	*D*—H	H···*A*	*D*···*A*	*D*—H···*A*
N1—H2*N*···O1	0.91	1.89	2.799 (5)	172
N1—H1*N*···Br3	0.91	2.47	3.232 (5)	141
N2—H3*N*···Br1	1.00	2.67	3.438 (4)	133
N2—H3*N*···Br1^iii^	1.00	2.67	3.438 (4)	133
O1—H1*O*···Br2^viii^	0.87	2.34	3.200 (3)	172
C1—H1*A*···Br2^iv^	0.99	2.88	3.614 (4)	131
C1—H1*B*···Br1^iii^	0.99	3.00	3.703 (4)	129
C2—H2*B*···Br1^iii^	0.99	3.05	3.517 (4)	111
C2—H2*B*···Br3^viii^	0.99	2.82	3.525 (4)	129
C3—H3*A*···Br1^ix^	0.98	2.96	3.857 (4)	153
C3—H3*B*···Br3^x^	0.98	2.75	3.615 (6)	148

Symmetry codes: (iii) −*x*+2, −*y*+1, *z*; (iv) −*x*+1, −*y*+1, *z*; (viii) *x*, *y*−1/2, *z*−1/2; (ix) −*x*+2, −*y*+3/2, *z*−1/2; (x) *x*, *y*, *z*−1.

### Poly[1-methylpiperizine-1,4-diium [tri-µ-bromido-rubidium] hemihydrate] (II) Crystal data

**Table:**

(C_5_H_14_N_2_)[RbBr_3_]·0.5H_2_O	*D*_x_ = 2.284 Mg m^−^^3^
*M**_r_* = 436.39	Mo *K*α radiation, λ = 0.71073 Å
Orthorhombic, *A**m**m*2	Cell parameters from 2309 reflections
*a* = 13.477 (3) Å	θ = 3.0–27.4°
*b* = 9.5617 (19) Å	µ = 13.31 mm^−^^1^
*c* = 9.850 (2) Å	*T* = 93 K
*V* = 1269.3 (5) Å^3^	Prism, colourless
*Z* = 4	0.15 × 0.10 × 0.10 mm
*F*(000) = 820	

### Poly[1-methylpiperizine-1,4-diium [tri-µ-bromido-rubidium] hemihydrate] (II) Data collection

**Table:**

Rigaku Pilatus 200K CCD diffractometer	1244 reflections with *I* > 2σ(*I*)
ω scans	*R*_int_ = 0.037
Absorption correction: multi-scan (CrysalisPro; Rigaku, 2017)	θ_max_ = 25.3°, θ_min_ = 3.0°
*T*_min_ = 0.347, *T*_max_ = 1.000	*h* = −16→16
6851 measured reflections	*k* = −11→11
1264 independent reflections	*l* = −11→11

### Poly[1-methylpiperizine-1,4-diium [tri-µ-bromido-rubidium] hemihydrate] (II) Refinement

**Table:**

Refinement on *F*^2^	H-atom parameters constrained
Least-squares matrix: full	*w* = 1/[σ^2^(*F*_o_^2^) + (0.0076*P*)^2^] where *P* = (*F*_o_^2^ + 2*F*_c_^2^)/3
*R*[*F*^2^ > 2σ(*F*^2^)] = 0.013	(Δ/σ)_max_ < 0.001
*wR*(*F*^2^) = 0.028	Δρ_max_ = 0.27 e Å^−^^3^
*S* = 1.01	Δρ_min_ = −0.35 e Å^−^^3^
1264 reflections	Extinction correction: SHELXL-2014/7 (Sheldrick 2015), Fc^*^=kFc[1+0.001xFc^2^λ^3^/sin(2θ)]^-1/4^
69 parameters	Extinction coefficient: 0.00327 (14)
1 restraint	Absolute structure: Parsons *et al.* (2013)
Primary atom site location: structure-invariant direct methods	Absolute structure parameter: −0.001 (13)
Hydrogen site location: mixed	

### Poly[1-methylpiperizine-1,4-diium [tri-µ-bromido-rubidium] hemihydrate] (II) Special details

**Table:**

Geometry. All esds (except the esd in the dihedral angle between two l.s. planes) are estimated using the full covariance matrix. The cell esds are taken into account individually in the estimation of esds in distances, angles and torsion angles; correlations between esds in cell parameters are only used when they are defined by crystal symmetry. An approximate (isotropic) treatment of cell esds is used for estimating esds involving l.s. planes.

### Poly[1-methylpiperizine-1,4-diium [tri-µ-bromido-rubidium] hemihydrate] (II) Fractional atomic coordinates and isotropic or equivalent isotropic displacement parameters (Å^2^)

**Table:**

	*x*	*y*	*z*	*U*_iso_*/*U*_eq_	
Rb1	0.0000	0.5000	0.09734 (7)	0.01032 (18)	
Rb2	0.5000	0.5000	−0.03670 (8)	0.00968 (16)	
Br1	0.0000	0.29829 (5)	0.39777 (6)	0.01090 (14)	
Br2	0.5000	0.25694 (6)	0.21977 (6)	0.01073 (13)	
Br3	0.25385 (4)	0.5000	0.03510 (6)	0.01017 (13)	
C1	0.2708 (3)	0.6293 (4)	0.3838 (4)	0.0124 (8)	
H1A	0.2118	0.6366	0.3243	0.015*	
H1B	0.3130	0.7123	0.3674	0.015*	
C2	0.2378 (3)	0.6284 (4)	0.5303 (5)	0.0117 (8)	
H2A	0.2966	0.6293	0.5905	0.014*	
H2B	0.1980	0.7131	0.5495	0.014*	
C3	0.1383 (4)	0.5000	0.6996 (5)	0.0149 (12)	
H3A	0.0985	0.4149	0.7150	0.022*	
H3B	0.1945	0.5000	0.7635	0.022*	
N1	0.3274 (3)	0.5000	0.3486 (5)	0.0107 (10)	
H1N	0.3862	0.5000	0.3941	0.013*	
H2N	0.3410	0.5000	0.2581	0.013*	
N2	0.1766 (3)	0.5000	0.5584 (4)	0.0087 (9)	
H3N	0.1187	0.5000	0.4950	0.010*	
O1	0.5000	0.5000	0.5097 (5)	0.0109 (12)	
H1O	0.5000	0.5749	0.5607	0.013*	

### Poly[1-methylpiperizine-1,4-diium [tri-µ-bromido-rubidium] hemihydrate] (II) Atomic displacement parameters (Å^2^)

**Table:**

	*U*^11^	*U*^22^	*U*^33^	*U*^12^	*U*^13^	*U*^23^
Rb1	0.0111 (4)	0.0093 (4)	0.0105 (4)	0.000	0.000	0.000
Rb2	0.0114 (4)	0.0091 (3)	0.0086 (3)	0.000	0.000	0.000
Br1	0.0122 (3)	0.0083 (3)	0.0122 (3)	0.000	0.000	0.0006 (2)
Br2	0.0141 (3)	0.0087 (3)	0.0094 (3)	0.000	0.000	0.0001 (2)
Br3	0.0112 (3)	0.0092 (3)	0.0101 (3)	0.000	−0.0012 (2)	0.000
C1	0.013 (2)	0.0077 (19)	0.017 (2)	−0.0003 (15)	−0.0003 (17)	0.0023 (18)
C2	0.013 (2)	0.0054 (18)	0.017 (2)	0.0009 (15)	0.0016 (18)	−0.001 (2)
C3	0.019 (3)	0.021 (3)	0.005 (3)	0.000	0.004 (2)	0.000
N1	0.011 (2)	0.012 (2)	0.009 (2)	0.000	0.0025 (19)	0.000
N2	0.009 (2)	0.010 (2)	0.007 (2)	0.000	−0.0010 (18)	0.000
O1	0.016 (3)	0.006 (2)	0.011 (3)	0.000	0.000	0.000

### Poly[1-methylpiperizine-1,4-diium [tri-µ-bromido-rubidium] hemihydrate] (II) Geometric parameters (Å, º)

**Table:**

Rb1—Br1^i^	3.4639 (8)	C1—C2	1.510 (6)
Rb1—Br1^ii^	3.4639 (8)	C1—H1A	0.9900
Rb1—Br1^iii^	3.5323 (10)	C1—H1B	0.9900
Rb1—Br1	3.5323 (10)	C2—N2	1.504 (4)
Rb1—Br3^iii^	3.4756 (9)	C2—H2A	0.9900
Rb1—Br3	3.4756 (9)	C2—H2B	0.9900
Rb2—Br2^iv^	3.4326 (9)	C3—N2	1.484 (6)
Rb2—Br2	3.4326 (9)	C3—H3A	0.9868
Rb2—Br2^v^	3.4336 (9)	C3—H3B	0.9852
Rb2—Br2^ii^	3.4336 (9)	N1—C1^vii^	1.494 (4)
Rb2—Br3	3.3919 (9)	N1—H1N	0.9100
Rb2—Br3^iv^	3.3920 (9)	N1—H2N	0.9100
Br1—Rb1^vi^	3.4639 (8)	N2—C2^vii^	1.504 (4)
Br2—Rb2^vi^	3.4336 (9)	N2—H3N	1.0000
C1—N1	1.494 (4)	O1—H1O	0.8748
			
Br1^i^—Rb1—Br1^ii^	110.85 (3)	Rb1^vi^—Br1—Rb1	157.67 (2)
Br1^i^—Rb1—Br3^iii^	84.255 (10)	Rb2—Br2—Rb2^vi^	176.93 (2)
Br1^ii^—Rb1—Br3^iii^	84.255 (10)	Rb2—Br3—Rb1	178.12 (3)
Br1^i^—Rb1—Br3	84.255 (10)	N1—C1—C2	111.6 (3)
Br1^ii^—Rb1—Br3	84.255 (10)	N1—C1—H1A	109.3
Br3^iii^—Rb1—Br3	159.68 (3)	C2—C1—H1A	109.3
Br1^i^—Rb1—Br1^iii^	157.67 (2)	N1—C1—H1B	109.3
Br1^ii^—Rb1—Br1^iii^	91.481 (16)	C2—C1—H1B	109.3
Br3^iii^—Rb1—Br1^iii^	98.498 (12)	H1A—C1—H1B	108.0
Br3—Rb1—Br1^iii^	98.498 (12)	N2—C2—C1	110.0 (3)
Br1^i^—Rb1—Br1	91.481 (15)	N2—C2—H2A	109.7
Br1^ii^—Rb1—Br1	157.67 (2)	C1—C2—H2A	109.7
Br3^iii^—Rb1—Br1	98.498 (12)	N2—C2—H2B	109.7
Br3—Rb1—Br1	98.498 (12)	C1—C2—H2B	109.7
Br1^iii^—Rb1—Br1	66.19 (3)	H2A—C2—H2B	108.2
Br3—Rb2—Br3^iv^	155.93 (3)	N2—C3—H3A	109.5
Br3—Rb2—Br2^iv^	81.173 (14)	N2—C3—H3B	109.4
Br3^iv^—Rb2—Br2^iv^	81.173 (13)	H3A—C3—H3B	108.6
Br3—Rb2—Br2	81.172 (13)	C1—N1—C1^vii^	111.7 (4)
Br3^iv^—Rb2—Br2	81.173 (14)	C1—N1—H1N	109.3
Br2^iv^—Rb2—Br2	85.22 (3)	C1^vii^—N1—H1N	109.3
Br3—Rb2—Br2^v^	98.376 (11)	C1—N1—H2N	109.3
Br3^iv^—Rb2—Br2^v^	98.376 (11)	C1^vii^—N1—H2N	109.3
Br2^iv^—Rb2—Br2^v^	176.93 (2)	H1N—N1—H2N	107.9
Br2—Rb2—Br2^v^	91.702 (16)	C3—N2—C2	111.3 (3)
Br3—Rb2—Br2^ii^	98.376 (11)	C3—N2—C2^vii^	111.3 (3)
Br3^iv^—Rb2—Br2^ii^	98.376 (11)	C2—N2—C2^vii^	109.4 (4)
Br2^iv^—Rb2—Br2^ii^	91.702 (16)	C3—N2—H3N	108.3
Br2—Rb2—Br2^ii^	176.93 (2)	C2—N2—H3N	108.3
Br2^v^—Rb2—Br2^ii^	91.37 (3)	C2^vii^—N2—H3N	108.3
			
N1—C1—C2—N2	−56.8 (4)	C1—C2—N2—C3	−176.8 (3)
C2—C1—N1—C1^vii^	53.6 (5)	C1—C2—N2—C2^vii^	59.8 (5)

Symmetry codes: (i) −*x*, −*y*+1/2, *z*−1/2; (ii) *x*, *y*+1/2, *z*−1/2; (iii) −*x*, −*y*+1, *z*; (iv) −*x*+1, −*y*+1, *z*; (v) −*x*+1, −*y*+1/2, *z*−1/2; (vi) *x*, *y*−1/2, *z*+1/2; (vii) *x*, −*y*+1, *z*.

### Poly[1-methylpiperizine-1,4-diium [tri-µ-bromido-rubidium] hemihydrate] (II) Hydrogen-bond geometry (Å, º)

**Table:**

*D*—H···*A*	*D*—H	H···*A*	*D*···*A*	*D*—H···*A*
N1—H1*N*···O1	0.91	1.91	2.816 (5)	173
N1—H2*N*···Br3	0.91	2.49	3.243 (4)	140
N2—H3*N*···Br1	1.00	2.68	3.448 (4)	134
N2—H3*N*···Br1^iii^	1.00	2.68	3.448 (4)	134
O1—H1*O*···Br2^viii^	0.87	2.34	3.212 (3)	173
C1—H1*A*···Br1^iii^	0.99	3.01	3.717 (4)	129
C1—H1*B*···Br2^iv^	0.99	2.92	3.652 (4)	131
C2—H2*B*···Br1^iii^	0.99	3.06	3.531 (4)	111
C2—H2*B*···Br3^viii^	0.99	2.85	3.560 (4)	130
C3—H3*A*···Br1^ix^	0.99	3.03	3.927 (4)	152
C3—H3*B*···Br3^x^	0.99	2.79	3.653 (5)	146

Symmetry codes: (iii) −*x*, −*y*+1, *z*; (iv) −*x*+1, −*y*+1, *z*; (viii) *x*, *y*+1/2, *z*+1/2; (ix) −*x*, −*y*+1/2, *z*+1/2; (x) *x*, *y*, *z*+1.

### Poly[1-methylpiperizine-1,4-diium [tri-µ-bromido-caesium] hemihydrate] (III) Crystal data

**Table:**

(C_5_H_14_N_2_)[CsBr_3_]·0.5H_2_O	*D*_x_ = 2.435 Mg m^−^^3^
*M**_r_* = 483.83	Mo *K*α radiation, λ = 0.71073 Å
Orthorhombic, *A**m**m*2	Cell parameters from 2407 reflections
*a* = 13.610 (3) Å	θ = 2.9–27.5°
*b* = 9.7201 (19) Å	µ = 11.85 mm^−^^1^
*c* = 9.977 (2) Å	*T* = 93 K
*V* = 1319.9 (5) Å^3^	Block, colourless
*Z* = 4	0.10 × 0.10 × 0.10 mm
*F*(000) = 892	

### Poly[1-methylpiperizine-1,4-diium [tri-µ-bromido-caesium] hemihydrate] (III) Data collection

**Table:**

Rigaku Pilatus 200K CCD diffractometer	1293 reflections with *I* > 2σ(*I*)
ω scans	*R*_int_ = 0.022
Absorption correction: multi-scan (CrysalisPro; Rigaku, 2017)	θ_max_ = 25.4°, θ_min_ = 2.9°
*T*_min_ = 0.463, *T*_max_ = 1.000	*h* = −16→15
3718 measured reflections	*k* = −11→11
1312 independent reflections	*l* = −11→12

### Poly[1-methylpiperizine-1,4-diium [tri-µ-bromido-caesium] hemihydrate] (III) Refinement

**Table:**

Refinement on *F*^2^	H-atom parameters constrained
Least-squares matrix: full	*w* = 1/[σ^2^(*F*_o_^2^) + (0.0012*P*)^2^] where *P* = (*F*_o_^2^ + 2*F*_c_^2^)/3
*R*[*F*^2^ > 2σ(*F*^2^)] = 0.013	(Δ/σ)_max_ < 0.001
*wR*(*F*^2^) = 0.029	Δρ_max_ = 0.57 e Å^−^^3^
*S* = 0.94	Δρ_min_ = −0.70 e Å^−^^3^
1312 reflections	Extinction correction: SHELXL-2014/7 (Sheldrick 2015), Fc^*^=kFc[1+0.001xFc^2^λ^3^/sin(2θ)]^-1/4^
68 parameters	Extinction coefficient: 0.00081 (7)
1 restraint	Absolute structure: Parsons *et al.* (2013)
Primary atom site location: structure-invariant direct methods	Absolute structure parameter: 0.016 (7)
Hydrogen site location: mixed	

### Poly[1-methylpiperizine-1,4-diium [tri-µ-bromido-caesium] hemihydrate] (III) Special details

**Table:**

Geometry. All esds (except the esd in the dihedral angle between two l.s. planes) are estimated using the full covariance matrix. The cell esds are taken into account individually in the estimation of esds in distances, angles and torsion angles; correlations between esds in cell parameters are only used when they are defined by crystal symmetry. An approximate (isotropic) treatment of cell esds is used for estimating esds involving l.s. planes.

### Poly[1-methylpiperizine-1,4-diium [tri-µ-bromido-caesium] hemihydrate] (III) Fractional atomic coordinates and isotropic or equivalent isotropic displacement parameters (Å^2^)

**Table:**

	*x*	*y*	*z*	*U*_iso_*/*U*_eq_	
Cs1	0.0000	0.5000	0.10292 (5)	0.01104 (16)	
Cs2	0.5000	0.5000	−0.04771 (5)	0.01285 (16)	
Br1	0.0000	0.30234 (7)	0.40657 (7)	0.01341 (18)	
Br2	0.5000	0.26080 (7)	0.21347 (7)	0.01513 (17)	
Br3	0.25365 (6)	0.5000	0.03902 (7)	0.01354 (16)	
C1	0.2714 (4)	0.6276 (5)	0.3848 (5)	0.0140 (11)	
H1A	0.2128	0.6348	0.3263	0.017*	
H1B	0.3132	0.7093	0.3686	0.017*	
C2	0.2391 (4)	0.6259 (4)	0.5301 (6)	0.0138 (10)	
H2A	0.2977	0.6263	0.5890	0.017*	
H2B	0.2001	0.7095	0.5497	0.017*	
C3	0.1412 (5)	0.5000	0.6985 (7)	0.0189 (16)	
H3A	0.1014	0.4149	0.7140	0.028*	
H3B	0.1974	0.5000	0.7625	0.028*	
N1	0.3276 (4)	0.5000	0.3497 (6)	0.0125 (13)	
H1N	0.3408	0.5000	0.2603	0.015*	
H2N	0.3859	0.5000	0.3944	0.015*	
N2	0.1782 (4)	0.5000	0.5586 (6)	0.0112 (12)	
H3N	0.1205	0.5000	0.4965	0.013*	
O1	0.5000	0.5000	0.5110 (6)	0.0149 (16)	
H1O	0.5000	0.5749	0.5620	0.018*	

### Poly[1-methylpiperizine-1,4-diium [tri-µ-bromido-caesium] hemihydrate] (III) Atomic displacement parameters (Å^2^)

**Table:**

	*U*^11^	*U*^22^	*U*^33^	*U*^12^	*U*^13^	*U*^23^
Cs1	0.0133 (4)	0.0096 (3)	0.0102 (3)	0.000	0.000	0.000
Cs2	0.0146 (3)	0.0134 (3)	0.0105 (3)	0.000	0.000	0.000
Br1	0.0155 (4)	0.0110 (3)	0.0138 (3)	0.000	0.000	0.0029 (3)
Br2	0.0193 (4)	0.0128 (3)	0.0133 (3)	0.000	0.000	−0.0001 (3)
Br3	0.0143 (3)	0.0140 (3)	0.0123 (4)	0.000	−0.0017 (3)	0.000
C1	0.015 (3)	0.009 (2)	0.018 (3)	0.001 (2)	0.001 (2)	0.003 (2)
C2	0.017 (3)	0.009 (2)	0.016 (2)	0.002 (2)	0.000 (2)	0.001 (3)
C3	0.021 (4)	0.026 (4)	0.009 (3)	0.000	0.003 (4)	0.000
N1	0.010 (3)	0.016 (3)	0.012 (3)	0.000	0.001 (2)	0.000
N2	0.012 (3)	0.012 (3)	0.010 (3)	0.000	0.001 (2)	0.000
O1	0.022 (4)	0.007 (3)	0.016 (4)	0.000	0.000	0.000

### Poly[1-methylpiperizine-1,4-diium [tri-µ-bromido-caesium] hemihydrate] (III) Geometric parameters (Å, º)

**Table:**

Cs1—Br1^i^	3.5319 (9)	C1—C2	1.515 (7)
Cs1—Br1^ii^	3.5319 (9)	C1—H1A	0.9900
Cs1—Br1^iii^	3.5873 (10)	C1—H1B	0.9900
Cs1—Br1	3.5873 (10)	C2—N2	1.505 (6)
Cs1—Br3^iii^	3.5105 (11)	C2—H2A	0.9900
Cs1—Br3	3.5105 (11)	C2—H2B	0.9900
Cs2—Br2^iv^	3.4923 (10)	C3—N2	1.484 (9)
Cs2—Br2	3.4923 (10)	C3—H3A	1.0011
Cs2—Br2^v^	3.4790 (9)	C3—H3B	0.9961
Cs2—Br2^ii^	3.4790 (9)	N1—C1^vii^	1.499 (6)
Cs2—Br3	3.4627 (11)	N1—H1N	0.9100
Cs2—Br3^iv^	3.4627 (11)	N1—H2N	0.9100
Br1—Cs1^vi^	3.5319 (9)	N2—C2^vii^	1.505 (6)
Br2—Cs2^vi^	3.4790 (9)	N2—H3N	1.0000
C1—N1	1.499 (6)	O1—H1O	0.8883
			
Br3^iii^—Cs1—Br3	159.07 (3)	Cs1^vi^—Br1—Cs1	156.07 (2)
Br3^iii^—Cs1—Br1^i^	84.218 (9)	Cs2^vi^—Br2—Cs2	174.97 (3)
Br3—Cs1—Br1^i^	84.219 (9)	Cs2—Br3—Cs1	175.99 (3)
Br3^iii^—Cs1—Br1^ii^	84.218 (9)	N1—C1—C2	111.3 (4)
Br3—Cs1—Br1^ii^	84.219 (9)	N1—C1—H1A	109.4
Br1^i^—Cs1—Br1^ii^	112.63 (3)	C2—C1—H1A	109.4
Br3^iii^—Cs1—Br1^iii^	98.823 (12)	N1—C1—H1B	109.4
Br3—Cs1—Br1^iii^	98.822 (12)	C2—C1—H1B	109.4
Br1^i^—Cs1—Br1^iii^	156.07 (2)	H1A—C1—H1B	108.0
Br1^ii^—Cs1—Br1^iii^	91.305 (15)	N2—C2—C1	110.4 (4)
Br3^iii^—Cs1—Br1	98.823 (12)	N2—C2—H2A	109.6
Br3—Cs1—Br1	98.822 (13)	C1—C2—H2A	109.6
Br1^i^—Cs1—Br1	91.305 (15)	N2—C2—H2B	109.6
Br1^ii^—Cs1—Br1	156.07 (2)	C1—C2—H2B	109.6
Br1^iii^—Cs1—Br1	64.76 (3)	H2A—C2—H2B	108.1
Br3—Cs2—Br3^iv^	151.06 (3)	N2—C3—H3A	109.2
Br3—Cs2—Br2^v^	99.854 (11)	N2—C3—H3B	110.0
Br3^iv^—Cs2—Br2^v^	99.854 (11)	H3A—C3—H3B	108.5
Br3—Cs2—Br2^ii^	99.854 (11)	C1^vii^—N1—C1	111.7 (5)
Br3^iv^—Cs2—Br2^ii^	99.854 (11)	C1^vii^—N1—H1N	109.3
Br2^v^—Cs2—Br2^ii^	93.55 (3)	C1—N1—H1N	109.3
Br3—Cs2—Br2^iv^	79.254 (13)	C1^vii^—N1—H2N	109.3
Br3^iv^—Cs2—Br2^iv^	79.255 (13)	C1—N1—H2N	109.3
Br2^v^—Cs2—Br2^iv^	174.97 (3)	H1N—N1—H2N	107.9
Br2^ii^—Cs2—Br2^iv^	91.485 (16)	C3—N2—C2	111.4 (4)
Br3—Cs2—Br2	79.254 (13)	C3—N2—C2^vii^	111.4 (4)
Br3^iv^—Cs2—Br2	79.255 (13)	C2—N2—C2^vii^	108.8 (5)
Br2^v^—Cs2—Br2	91.485 (16)	C3—N2—H3N	108.4
Br2^ii^—Cs2—Br2	174.97 (3)	C2—N2—H3N	108.4
Br2^iv^—Cs2—Br2	83.48 (3)	C2^vii^—N2—H3N	108.4
			
N1—C1—C2—N2	−57.1 (6)	C1—C2—N2—C3	−176.9 (4)
C2—C1—N1—C1^vii^	53.6 (7)	C1—C2—N2—C2^vii^	60.0 (7)

Symmetry codes: (i) −*x*, −*y*+1/2, *z*−1/2; (ii) *x*, *y*+1/2, *z*−1/2; (iii) −*x*, −*y*+1, *z*; (iv) −*x*+1, −*y*+1, *z*; (v) −*x*+1, −*y*+1/2, *z*−1/2; (vi) *x*, *y*−1/2, *z*+1/2; (vii) *x*, −*y*+1, *z*.

### Poly[1-methylpiperizine-1,4-diium [tri-µ-bromido-caesium] hemihydrate] (III) Hydrogen-bond geometry (Å, º)

**Table:**

*D*—H···*A*	*D*—H	H···*A*	*D*···*A*	*D*—H···*A*
N1—H2*N*···O1	0.91	1.94	2.845 (7)	173
N1—H1*N*···Br3	0.91	2.51	3.259 (6)	140
N2—H3*N*···Br1	1.00	2.68	3.446 (5)	134
N2—H3*N*···Br1^iii^	1.00	2.68	3.446 (5)	134
O1—H1*O*···Br2^viii^	0.89	2.36	3.242 (4)	175
C1—H1*A*···Br1^iii^	0.99	3.07	3.762 (5)	128
C1—H1*B*···Br2^iv^	0.99	2.99	3.712 (5)	131
C2—H2*B*···Br1^iii^	0.99	3.08	3.550 (5)	111
C2—H2*B*···Br3^viii^	0.99	2.92	3.643 (4)	131
C3—H3*B*···Br3^ix^	1.00	2.86	3.726 (7)	145

Symmetry codes: (iii) −*x*, −*y*+1, *z*; (iv) −*x*+1, −*y*+1, *z*; (viii) *x*, *y*+1/2, *z*+1/2; (ix) *x*, *y*, *z*+1.
